# Selected Methods and Applications of Anti-Friction and Anti-Wear Surface Texturing

**DOI:** 10.3390/ma14123227

**Published:** 2021-06-11

**Authors:** Slawomir Wos, Waldemar Koszela, Pawel Pawlus

**Affiliations:** Faculty of Mechanical Engineering and Aeronautics, Rzeszow University of Technology, Powstańców Warszawy 8, 35-959 Rzeszów, Poland; wosslawomir@prz.edu.pl (S.W.); wkktmiop@prz.edu.pl (W.K.)

**Keywords:** friction reduction, surface texturing, plastic deformation, abrasive jet machining

## Abstract

The constant development of environmental protection causes the necessity to increase the efficiency of machines. By increasing the efficiency of machines, energy losses can be limited, leading to lower energy consumption. Friction reduction leads to an increase in efficiency and a decrease in wear. In this paper, selected surface texturing methods, such as burnishing and abrasive jet machining, with their limitations are presented. Thanks to those processes, various surface textures can be obtained. Examples of applications of these methods for friction and wear reduction are shown.

## 1. Introduction

Surface texturing, due to the creation of dimples acting as reservoirs of lubricant in the contact zone, leads to an increase in wear resistance and to a change in friction from dry or boundary to mixed or full film lubrication. Dimples manufactured on frictional surfaces can also act as traps for wear debris, reducing wear. With special-shaped oil pockets and a sufficient amount of lubricant, surface texturing can cause the generation of a hydrodynamic lift, leading to a decrease in the coefficient of friction. By the proper selection of texture parameters such as oil pocket shape and depth, oil consumption, and pit-area ratio, a friction reduction effect can be obtained [1,2,3,4,5,6].

There are many texturing techniques [7]. Laser surface texturing [2,8,9,10] is the most common method of dimple creation. This technique is precise and allows for the easy creation of dimples. However, surface heating by the laser beam can lead to the creation of a heat-affected zone. In some cases, this disadvantage is unacceptable, especially for coated sliding elements. In most cases, after laser texturing, burrs occur around oil pockets and additional operation is required to remove them. Other techniques, such as burnishing (embossing) [11], mechanical polishing, milling [12], etching [13], abrasive jet machining [14] or a combination of these methods [15], can be used for dimple creation. It is not difficult to create oil pockets of comparatively large dimensions, applied, for example, in the sleeves of slide bearings, contrary to micro-dimples formed on thin-walled or coated elements.

In this work, two methods of dimple creation by burnishing and abrasive jet machining developed by the authors will be presented. In the surface layer after burnishing and abrasive jet machining, compressive residual stresses are created. They have positive effects on wear resistance and fatigue strength. These methods are supplements to laser surface texturing. They can be used for critical elements affecting the safety of construction where laser treatment was not approved. There can be problems with the creation of deep dimples by the burnishing of thin-walled elements, in contrast to abrasive jet machining. The selected tribological applications of these techniques will be given.

## 2. Selected Methods of Surface Texturing

### 2.1. Burnishing

**Figure 1:**
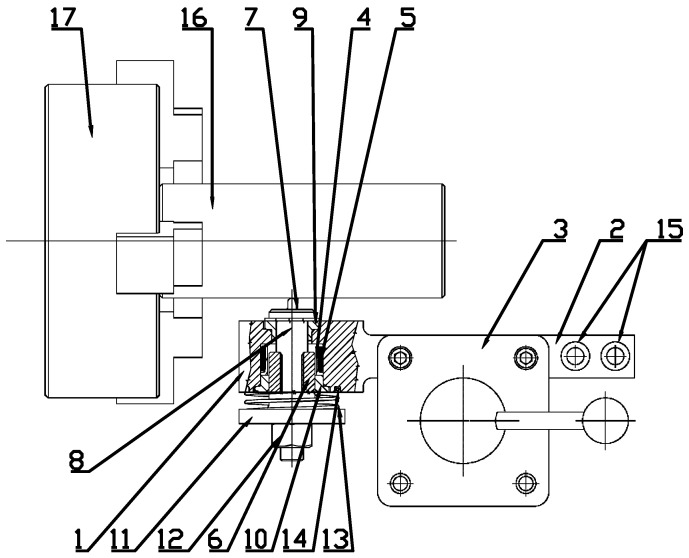
Scheme of device for surface texturing by burnishing technique.

The burnishing process changes the machined surface by plastic deformation. Dimple size and shape result from the shape of the tool. Therefore, dimple diameter can be smaller after using laser surface texturing. The depth of the dimples depends on the hardness of the machined material and on the kinetic energy of the moving elements of the burnishing head. The risk of surface damage is low when the burnishing process is performed correctly. Balls of different sizes, cones and Vickers hardness tips can be used as tools. Figure 1 presents the device for dimple creation by burnishing [16].

This device consists of an electromagnetic coil which is the controllable propulsion for the ferromagnetic core.

The tool for forming micro-oil pockets consists of casing (1) with a fixture (2) that was mounted in a tool holder (3) of a lathe not visible in Figure 1. Inside the tool slot (4) is a ferromagnetic coil (5), which is a propulsion and drive for the ferromagnetic core (6) and its forming tip. The core (6) and the pivot (8) were moving inside the driving tubes (9) and (10) mounted inside the casing (1). On the opposite side of the pivot (8), there is a pad (11) maintained by a nut (12). Between the pad (11) and the casing (1), there is a spring (13) supported by a fragment of the casing (1). At the fixture (2) of the casing (1), there are mounted clamps (15) used for powering the coil (5) by the electronic controller.

The oil pocket forming process is similar to standard lathing. However, instead of the cutting tool, a forming tip (7) was used, which was constantly hitting the textured surface. For obtaining a sufficient energy impulse, the distance between the forming tip (7) and the machined surface (16) is very important.

This distance should be determined when the core (6) is completely hidden inside the coil (5). The impact energy is lowest for the small distance between the forming tip and the machined surface. During the forming process, the possibility of changing the impact energy exists, which corresponds to the size of the received oil pockets. Such changes can be stepwise or gradual. The forming process will start when sufficient voltage powers the coil (5), producing the force that propels the core (6). The pattern of the obtained texture depends on the parameter set of the lathe, such as rotational speed and the feed, as well as the settings of the coil controller. Creation of the front surface requires a change in the mounting device in the tool holder (3), so this device will be perpendicular to the axis (17) of the lathe spindle.

In addition, different mechanisms for pressure generation can be used. An electromagnet that generates the reciprocating movement of a mounted tool can be an example [17].

**Figure 2:**
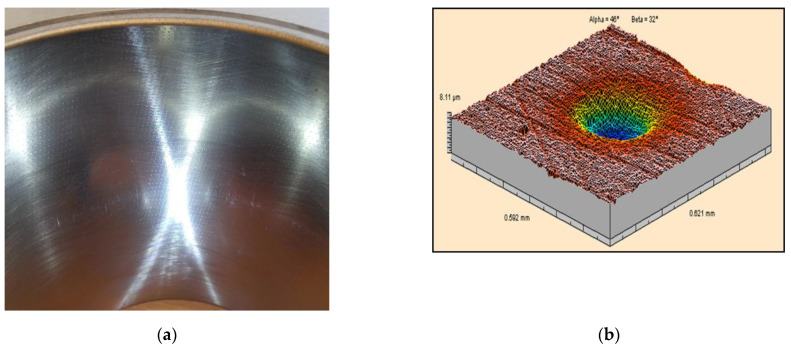
View of Nikasil cylinder liner (**a**) and topography of the single dimple (**b**).

Ceramic forming elements were used for dimple creation on cylinders made of cast iron steel balls and for cylinders made of Nikasil (Figure 2).

There are the following advantages of surface texturing by burnishing:

- No heat-affected zone around the dimples;
- The layers covering formed surface are not thinned, just pressed into the base material without affecting layer integrity;
- Fast process.

There is the following disadvantage:

- Positioning of the tool and the machined surface requires great precision because it affects the depth of the dimples.

### 2.2. Abrasive Jet Machining

**Figure 3:**
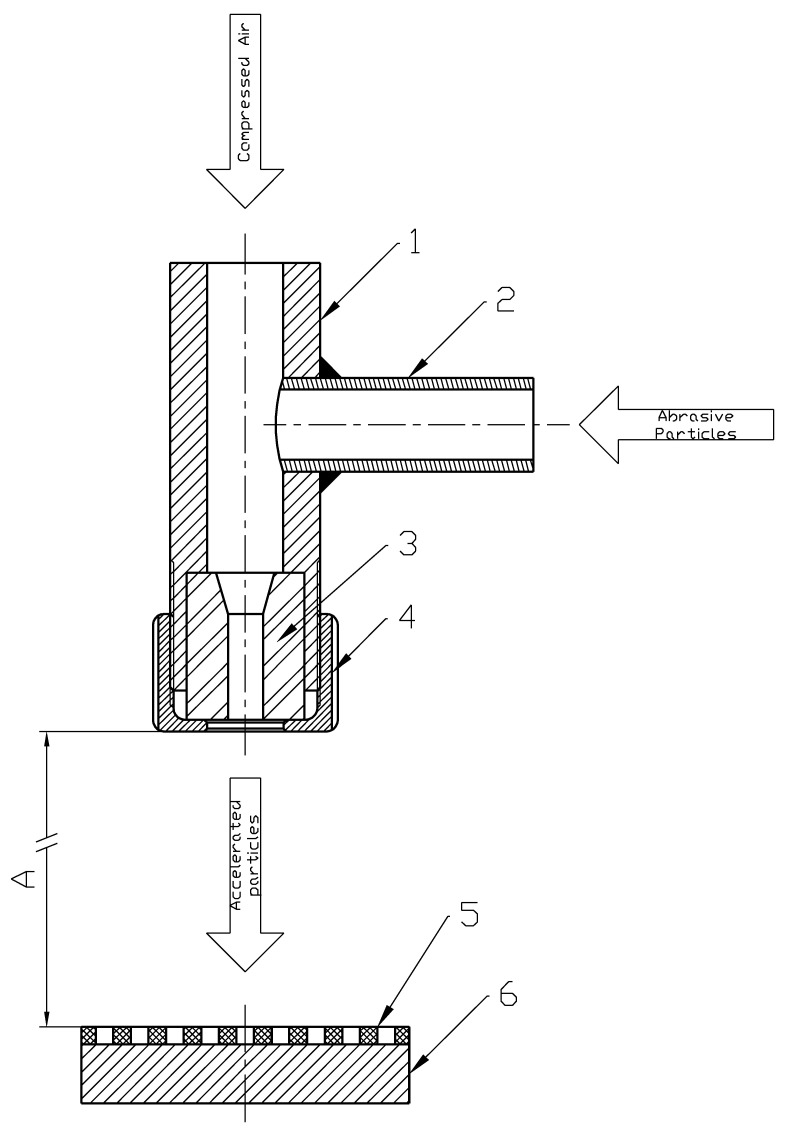
Scheme of surface texturing by abrasive jet machining method.

In this technique, the erosion process caused by speeded abrasive particles was used [18]. Such an approach can be beneficial when materials are highly resistant to temperature or are heat sensitive. Abrasive jet machining can be used for all types of materials; however, process parameters and used abrasive particles must be selected adequately for the textured material. The first use of this method was conducted with the use of a single small micro nozzle. This method, however, could only be used to machine one dimple at a time. This approach was much less effective than laser surface texturing due to the longer exposure time to make one dimple. In the method presented in this article, a different approach is introduced. Instead of using a single micro nozzle, a larger scale injector sandblaster was applied. However, to obtain dimples of a small size and a large number of samples, a masking technique was used [19]: a mask protects the machined surface which should not be exposed to the erosive influence of accelerated particles. Moreover, the overall shape of the dimples is a reflection of the holes made inside the mask. Further, because of the use of a bigger-than-micro nozzle typical sandblaster, a large area with many dimples can be textured at the same time. The main principles of this method are presented in Figure 3. An injector sandblaster is used in this process, where particles are accelerated by compressed air connected with a reinforced hose to the injector pistol (1). Typically perpendicular to the air flow direction, a second hose (2) is connected, and its end is inside the abrasive particle container. Inside the injector pistol, an area under pressure is generated which sucks the abrasive particles. Then, the particles go to the ceramic nozzle (3) held by a shaped nut (4). This nozzle causes increasing pressure and resulting speed of the particles. The mask (5) protects the surface which should not be machined. Dimension ‘A’ represents the distance between the nozzle and the machined material and should be adjusted experimentally according to the material type and hardness. In the presented case, an approximate distance of 100 mm was used. Two types of mask can be used. The first type of disposable mask is made of two layers of adhesive fiber glass hardened engraving foil, which can be used only once. The second type is made of a 0.3 mm thick sheet of metal and can be used multiple times, until the shape of perforation starts to deform due to erosion. For metal masks, the number of uses depends on the abrasive jet machining process parameters and on the level of acceptable differences between the assumed and real shapes.

This method can be applied to the production of flat textured areas on typical sandblasting stands. Typically, aluminum oxide is used for this purpose of grain size at least three times smaller than the dimple diameter. The operating pressure should be adjusted to the machined material.

**Figure 4:**
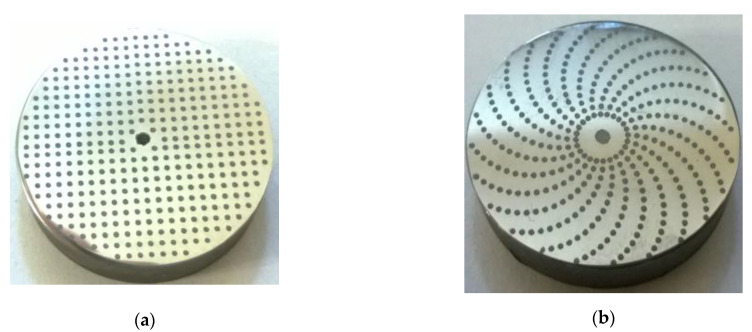
Examples of dimples and patterns made with abrasive jet machining, (**a**) square array, and (**b**) spiral array.

Similar to laser texturing, different shapes of dimples and their patterns can be created using abrasive jet machining (Figure 4). However, a spacing of at least 0.5 mm is needed to prevent distortion of the mask by the abrasive particles. Smaller distances between dimples can be achieved by laser surface texturing.

There are the following advantages of abrasive jet machining:

- No heat-affected zone around dimples;
- Fast process;
- The possibility of using multiple used masks;
- No burrs around the dimples;
- Nontoxic process;
- Multiple usage of the abrasive particles;
- Possibility of performing in a typical abrasive blasting stand.

There is the following disadvantage:

- Required precision in mask positioning.

## 3. Tribological Applications of Surface Texturing

### 3.1. The Effect of the Presence of Burnished Dimples on Wear Improvement

**Figure 5:**
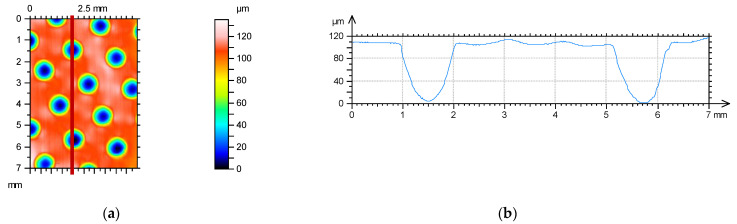
Contour plot (**a**) and profile (**b**) of textured surface.

The effect of surface texturing on linear wear was tested using a block-on-ring tester T-05 (Lukasiewicz Resarch Network - Institute for Sustainable Technologies, Radom, Poland). Dimples were created using the burnishing method on a stationary block surface from CuSn10P bronze after precision turning using the method described in patent [16]—Figure 1. This block co-acted with a rotating ring made of steel 42Crmo4 of 40 HRC hardness. The normal load during tests was constant, of 1500 N, and the rotational speed was set to 200 rpm, while the sliding speed was 0.22 m/s. The area of contact between the ring and the block was 100 mm^2^, and the contact pressure of untextured assembly was 15 MPa. The sliding distance was 5.06 km and the number of revolutions was 46,000. The friction assembly was lubricated with mineral oil L-AN46 (PKN ORLEN S.A., Poland). Tests were conducted in conformal sliding conditions. The oil capacities of the textured surfaces were 0.44, 0.55, and 0.93 mm^3^. The dimples had circular shapes. The roughness heights of the untextured sample and of the counter sample determined by the Ra parameter were 0.3 and 0.15 µm, respectively. Linear wear of the sliding pair: block-on-ring was measured after the sliding distance of: 220, 660, 1760, 2860, 3960 and 5060 m. The number of test repetitions was three. Figure 5 presents an example of a textured surface.

**Figure 6:**
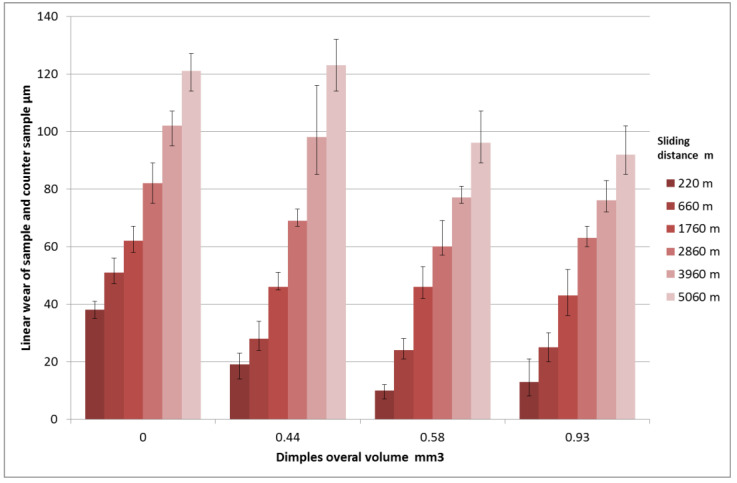
The effect of oil capacity of textured surface on linear wear of a tribological system.

Figure 6 presents the results of the linear wear of the tribological system. Wear was measured by an inductive sensor.

The beneficial effect of surface texturing on the tribological performance of the sliding pair was visible for the lowest sliding distance. The sample characterized by the medium oil capacity assured a decrease in linear wear of up to 3.8 times compared to the untextured one. Among the textured assemblies, the smallest oil capacity led to the highest linear wear. For the sliding distance of 660 m, the beneficial effect of surface texturing is still evident. However, surface texturing caused a decrease in the overall linear wear about two times, independently of the oil capacity. The differences between wear levels of untextured and textured assemblies were between 25 and 30 µm. When the sliding distances were 1760 and 2860 m, this difference decreased to about 20 µm. In these cases, surface texturing still improved the tribological performance of sliding assemblies. For the highest sliding distances of 3960 and 5060 m, the presence of dimples caused wear improvement only when the oil capacity was 0.58 or 0.93 mm^3^.

The wear values were related to the running-in. For the untextured pair, the running-in was finished when the sliding distance was 660 m. From this time, the wear intensity decreased. When the oil capacities were 0.58 or 0.95 mm^3^, the wear intensity slightly decreased when the sliding distance was higher than 1760 m. A different course of wear intensity was obtained for the sample with the smallest oil capacity (0.44 m^3^). Wear intensity versus time was the same when the sliding distances were up to 2860 m. When the sliding distance was larger, the wear intensity considerably increased. This increase was probably caused by oil pocket filling, which was caused by the smallest oil capacity. After this filling, wear debris got out from dimples to the contact zone, leading to increased wear.

One can see from this analysis that the beneficial effect of surface texturing was related to an increase in running-in time. Therefore, the best effects were obtained in the initial parts of the tests. Dimples were traps for wear debris leading to wear reduction. The sizes of the dimples should be chosen taking the operating conditions into account.

The effects of a textured surface created by the burnished technique on wear resistance of sliding elements in other investigations were presented in papers [20,21].

### 3.2. The Effect of Surface Texturing by Abrasive Jet Machining on Friction Reduction

**Figure 7:**
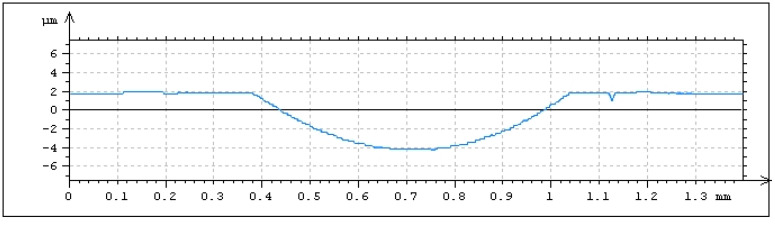
Profile with oil pocket made by abrasive jet machining.

**Figure 8:**
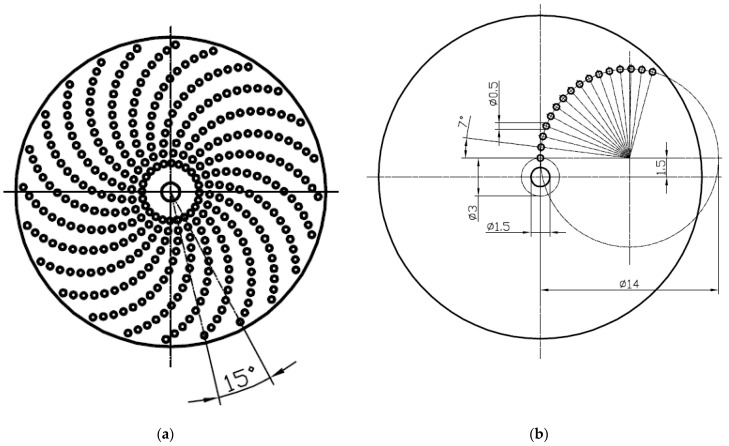
Drawing of the laser cut mask (**a**) and the dimensions of texture (**b**), all dimensions are in mm.

Samples prepared for abrasive jet machining using the device presented in Figure 2 were made of 42CrMo4 steel tempered to achieve a hardness of 40 ± 1 HRC. Discs of diameter 52.4 mm and of 8 mm thickness were samples. Before texturing, the contact surfaces of samples and counter samples were ground and polished to achieve the roughness described by the Ra parameter of 0.07 ± 0.01 µm. The untextured surface was also tested. A small disc with a diameter of 5 mm was the counter sample. The contact surfaces of the counter samples were chamfered 0.5 mm × 45° for better oil gathering capabilities. The counter samples were mounted on a holding pin, so the contact surface of the counter sample was self-aligned with the co-acting surface of the sample. The nominal contact areas of the sample and counter sample were rounded with diameters of 4 mm. Textured samples were processed with the use of an injector sandblaster; however, the particles used for the abrasive jet machining of aluminum oxide had 99.9% purity and grain size from 75 to 105 µm. Work pressure was set to 0.8 MPa, the nozzle diameter was 6 mm, and the distance between the surface and nozzle was approximately 100 mm. Exposure time was 30 s. To cover the non-machined surface details, a laser cut mask reinforced with fiber glass polypropylene was used. The overall shapes of the oil pockets were obtained through the projection of holes in the mask, and the depth of the oil pockets was controlled by the exposure time. Machined oil pockets were of a circular shape, of 0.5 mm diameter and of 9 ± 1 µm depth (Figure 7). Dimensions of the laser cut mask are shown in Figure 8.

**Figure 9:**
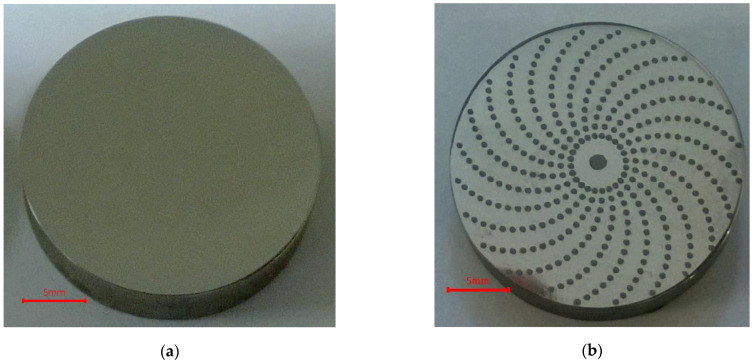
Photos of examples of (**a**) untextured and (**b**) textured discs.

The pit-area ratio of the tested samples was 15%, and dimples were in a spiral array (Figure 9).

**Figure 10:**
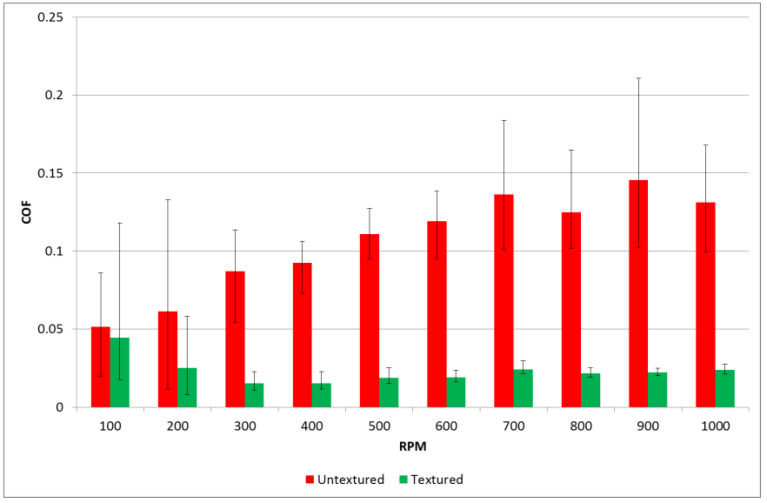
Average values and scatters of the coefficient of friction for textured and untextured samples at a normal load of 20 N.

**Figure 11:**
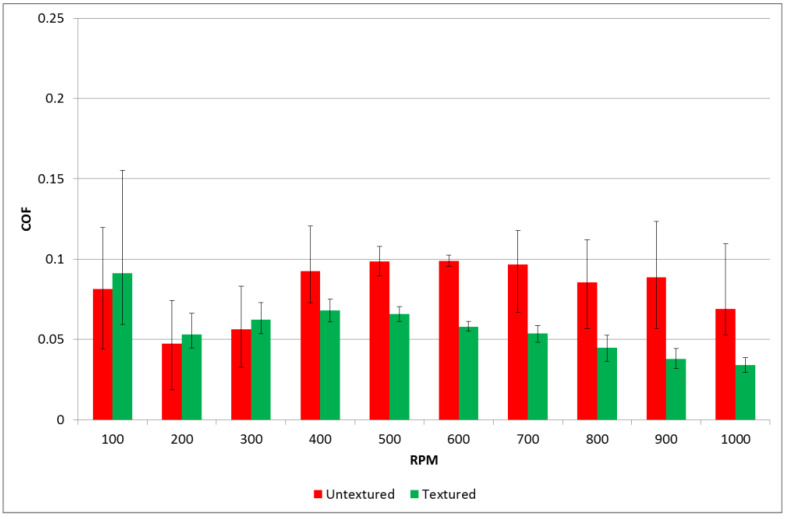
Average values and scatters of the coefficient of friction for textured and untextured samples at a normal load of 40 N.

Tests were carried out using a pin-on-disc tribotester T-11. Tests were performed with normal loads of 20 and 40 N. Rotational speed changed during the test from 100 to 1000 rpm with a step of 100 rpm. The test duration was 600 s, and the sliding distance was 276.5 m. The friction radius was 8 mm for all tests. The number of test repetitions was three. Figure 10 and Figure 11 show the average values of the coefficients of friction and scatters for various rotational speeds for loads of 20 and 40 N, respectively. The scatter was defined as the difference between the highest and the smallest coefficients of friction. Before each test, one drop of oil L-AN-46 was put into the inlet side of the contact zone. To achieve starved lubrication conditions, no additional oil was added during the test. There were the following lubricant parameters: kinematic viscosity at 40 °C: 46 mm^2^/s; ignition temperature was 235 °C; and the temperature of solidification was −15 °C.

Different results were achieved for various normal loads. When the load was lower, the coefficient of friction for the assembly with untextured samples increased as the test progressed. However, when the textured samples were tested, the friction force after the initial fluctuation initially decreased to about 0.02 and then increased with time. When the rotational speeds were 100 and 200 rpm, the differences between the coefficients of friction of sliding pairs with textured or untextured disc samples were insignificant. However, for higher speeds, surface texturing led to a much smaller coefficient of friction compared to untextured assembly; the highest ratio was near five. Furthermore, surface texturing caused much smaller variations of the coefficients of friction when the rotational speed was higher than 200 rpm.

When the normal load was higher, the runs of the coefficient of friction with time for assemblies with textured and untextured discs were similar. The coefficient of friction after the initial fluctuation decreased, then increased, obtained the maximum value and then slowly decreased. The beneficial effect of surface texturing was found only for rotational speed higher than 400 rpm. However, the maximum decrease in the coefficient of friction due to disc surface texturing was about two times.

An increase in the normal load caused a decrease in the coefficient of friction of the untextured assembly. This was probably caused by better matching of the contacting parts. However, when the textured disc samples were tested, an increase in the load caused an increase in the coefficient of friction. The better tribological behavior of the textured samples for the smaller normal load can be explained by the better possibility of creating additional hydrodynamic lift by dimples. An increase in the friction force with time in the last part of the test, when the normal load was smaller, can be explained by the lack of lubricant caused by the presence of centrifugal force. In this case, the contact between surfaces was smaller compared to the behavior under the larger load. When the normal load was larger, the oil was squeezed rather than delivered to the surface in contact for small sliding speeds. When the speed was higher, the squeeze effect decreased and the two surfaces were better matched; therefore, the coefficient of friction decreased.

Wear levels of the disc samples were negligible.

The beneficial effects of disc texturing by abrasive jet machining on the friction force were described in [22,23,24].

## 4. Conclusions

1. Burnishing changes the treated surface by plastic deformation. Dimple size and shape are the result of the shape of the tool. The burnishing process is fast. The zone near the dimples is not affected by heat. The positioning of the tool with regard to the treated surface needs high precision. Therefore, special devices for surface texturing by the burnishing process should be developed.
2. Abrasive jet machining is an interesting alternative to laser texturing. The erosion process caused by speeded abrasive particles was used. Abrasive jet machining can be applied for all types of materials. Similar to burnishing, this process is fast and the zone around the oil pockets is not influenced by heat. In addition, burrs around dimples were not created and multiple usages of masks and abrasives are possible. However, precision in the positioning of the masks is needed.
3. Surface texturing by burnishing of the block from bronze caused a decrease in the total linear wear of ring-on-block assembly up to four times. The beneficial effect of surface texturing was related to an increase in running-in time. This effect depends on the oil capacity. Dimples were traps for wear debris, leading to wear reduction. The dimensions of the dimples should be selected taking the operating conditions into consideration.
4. Disc surface texturing of the steel disc caused a decrease in the coefficient of friction of the pin-on-disc pair in unidirectional sliding up to five times. This beneficial effect was larger for higher speeds and smaller normal load. In addition, surface texturing led to lower variations of the coefficients of friction, especially for high sliding speeds.

## Data Availability

The data presented in this study are available on request from the corresponding author.
